# A Deep Learning-Based Detection and Segmentation System for Multimodal Ultrasound Images in the Evaluation of Superficial Lymph Node Metastases

**DOI:** 10.3390/jcm14061828

**Published:** 2025-03-08

**Authors:** Roxana Rusu-Both, Marius-Cristian Socaci, Adrian-Ionuț Palagos, Corina Buzoianu, Camelia Avram, Honoriu Vălean, Romeo-Ioan Chira

**Affiliations:** 1 Automation Department, Technical University of Cluj-Napoca, 400114 Cluj-Napoca, Romania; palagos.io.adrian@student.utcluj.ro (A.-I.P.); buzoianu.va.dana@campus.utcluj.ro (C.B.); honoriu.valean@aut.utcluj.ro (H.V.); 2 AIMed Soft Solution S.R.L., 400505 Cluj-Napoca, Romania; cristian.socaci@ai-med.ro; 3 Department of Internal Medicine, “Iuliu Hatieganu” University of Medicine and Pharmacy, 400347 Cluj-Napoca, Romania; romeochira@yahoo.com; 4 Gastroenterology Department, Emergency Clinical County Hospital Cluj-Napoca, 400347 Cluj-Napoca, Romania

**Keywords:** lymph node evaluation, ultrasound, deep learning, Mask R-CNN, segmentation, computer-aided diagnostic

## Abstract

**Background/Objectives**: Even with today’s advancements, cancer still represents a major cause of mortality worldwide. One important aspect of cancer progression that has a big impact on diagnosis, prognosis, and treatment plans is accurate lymph node metastasis evaluation. However, regardless of the imaging method used, this process is challenging and time-consuming. This research aimed to develop and validate an automatic detection and segmentation system for superficial lymph node evaluation based on multimodal ultrasound images, such as traditional B-mode, Doppler, and elastography, using deep learning techniques. **Methods**: The suggested approach incorporated a Mask R-CNN architecture designed specifically for the detection and segmentation of lymph nodes. The pipeline first involved noise reduction preprocessing, after which morphological and textural feature segmentation and analysis were performed. Vascularity and stiffness parameters were further examined in Doppler and elastography pictures. Metrics, including accuracy, mean average precision (mAP), and dice coefficient, were used to assess the system’s performance during training and validation on a carefully selected dataset of annotated ultrasound pictures. **Results**: During testing, the Mask R-CNN model showed an accuracy of 92.56%, a COCO AP score of 60.7 and a validation score of 64. Furter on, to improve diagnostic capabilities, Doppler and elastography data were added. This allowed for improved performance across several types of ultrasound images and provided thorough insights into the morphology, vascularity, and stiffness of lymph nodes. **Conclusions**: This paper offers a novel use of deep learning for automated lymph node assessment in ultrasound imaging. This system offers a dependable tool for doctors to evaluate lymph node metastases efficiently by fusing sophisticated segmentation techniques with multimodal image processing. It has the potential to greatly enhance patient outcomes and diagnostic accuracy.

## 1. Introduction

The societal impact of cancer is substantial worldwide. According to World Health Organization (WHO) data from 2022, there were around 9.7 million cancer-related deaths and 20 million new cancer diagnoses, with these figures being predicted to rise [1,2]. Tumor metastases are the main cause of cancer-related death. Regional lymph nodes (LNs) can be the site of early cancer metastases, playing a crucial role in cancer staging, treatment, and prognosis [3].

Tumor–node–metastasis (TNM) is a globally recognized standard for the classification of malignant tumors, with N representing the degree of the spread of the tumor in the LNs [4,5]. Accurate evaluation of the lymphatic extension of a tumor is essential, as patients with LN metastasis face a higher risk of recurrence or death from cancer. Besides cancer staging and prognosis, the precise identification and description of LN involvement are essential for treatment decisions, especially for tumors like head and neck squamous cell carcinoma (HNSCC), breast, lung, colon, and prostate [6]. Correct cancer staging directly impacts treatment decisions, including the need for preoperative therapy, adjuvant treatment, and the extent of surgery. Incorrect staging by the misclassification of LN involvement can lead to improper treatment choices, affecting both patients’ health outcomes and quality of life [7,8,9].

The gold standard for the assessment of LN is represented by LN excision, lymphadenectomy, followed by histopathological confirmation [10]. However, the surgical excision of LN is invasive, expensive and carries risks of developing certain complications, such as vascular and nerve injuries, edema, and cyst formation [10,11,12,13]. An alternative is represented by sentinel LN detection, biopsy, and removal [7]. Several imagistic methods like computed tomography (CT), magnetic resonance imaging (MRI), and positron emission tomography (PET-CT) can offer valuable anatomical and functional insights into LN metastases [14,15,16]. However, these techniques have disadvantages, such as exposure to ionizing radiation, high costs, and reduced sensitivity for small or superficial LNs [17]. Additionally, the availability of these imaging techniques is low, up to several months in some national healthcare programs, hindering timely diagnosis and treatment decisions [17]. Ultrasonography (US) can represent a promising alternative, being non-invasive, real-time, and cost-effective, and it offers superior spatial resolution, particularly for assessing superficial LNs. Doppler-mode US and elastography can further enhance diagnostic accuracy by assessing vascularity and tissue stiffness.

Despite the large number of advantages, US-based diagnosis remains highly operator-dependent, with accuracy varying based on physician experience and workload [18,19]. Often, less-experienced physicians or radiologists find it difficult to correctly identify LN and asses the morphology, vascular patters, or stiffness [18].

These challenges highlight the need for a more accurate, quantitative, non-invasive and cost-effective diagnostic support tool to assist clinicians in LN identification and characterization, ultimately improving treatment decisions.

Deep learning (DL) techniques have revolutionized medical imaging by providing reliable tools for the automatic detection, segmentation, and classification of complex anatomical structures. However, most DL studies focus on CT and PET-CT images, while ultrasound-based DL applications remain limited, primarily addressing the detection and evaluation of breast lesions and thyroid nodules [20,21,22,23,24,25,26,27]. Convolutional neural networks (CNNs), including U-Net architectures, have demonstrated remarkable success in segmenting LNs from ultrasound images. In [28], Li et al. propose a multi-task CNN for predicting LN metastasis and classifying tumors as benign or malignant in breast US images, with good results. Similarly, in [29], Sun et al. use a CNN with 12 convolutional layers for assessing LN metastasis using primary breast cancer images, achieving an accuracy of 72.6%, a sensitivity of 65.5%, and a specificity of 78.9%. In [24], Chen et al. present a U-Net convolutional neural network integrated with diffusion-based de-speckling for automatic and accurate LN segmentation, reporting 93.4% accuracy, 93.9% sensitivity, and 93.7% specificity.

For CT-based LN metastasis assessment, several CNN models such as U-net [30], AlexNet [31], DetectNet [32], and DualNet [33] have been explored, achieving an AUCs of 0.8 using AlexNet [31] and 0.95 using U-net [30].

The current work presents an automatic DL detection and segmentation system for LN metastasis evaluation using multimodal US data, including B-mode, Doppler, and elastography images. This approach aims to enhance diagnostic accuracy and reduce variability, integrating preprocessing techniques like denoising and edge enhancement, a Mask R-CNN model for segmentation, and additional image processing modules that perform computations to obtain relevant information about the vascularity, tissue hardness, shape, and contour definition of the analyzed LN.

The automation of LN analysis based on multimodal US images could lead to significant impact in clinical practice by diminishing the dependency on the operator experience, facilitating consistent and objective evaluations. Furthermore, this type of automated system offers a scalable approach that could enable early diagnosis, optimizing treatment strategies and eventually improving patient outcomes.

The use of multimodal US data represents an innovative approach for LN metastasis evaluation by integrating morphologic, vascular, and stiffness-related information, potentially setting a new reference.

Overall, the proposed solution has several potential advantages, being non-invasive, radiation-free, widely accessible, safe for long-term patient surveillance, and suitable for patient stratification. The solution also has a potential for the improvement of clinical decisions and cost-effectiveness.

By leveraging DL and multimodal imaging, this study aligns with precision oncology goals, contributing to early and accurate LN metastasis detection.

## 2. Materials and Methods

### 2.1. Study Design and Setting

Patient consent was waived due to the retrospective design of this study, based on pre-existing ultrasound images. However, the pre-existing images were obtained from a study performed in 2023, which, at that time, was approved by the County Emergency Hospital Cluj and other hospitals referred to the 1st Medical Clinic. Participation required informed consent as per the Declaration of Helsinki, with ethical approval from the UMF “Iuliu Hatieganu” Ethics Committee. All procedures (research protocol, data collections) conformed to ethical standards. The sample size (number of cases and ultrasound images) was determined by the number of patients that met the inclusion criteria during the study period, from July 2023 to December 2023. During the study period, consecutive patients with clinical or paraclinical/imaging suspicion of pathological adenopathy were enrolled, resulting in 65 cases (patients) and 506 US images of LNs, corresponding to specific LNs scanned from multiple LN stations per patient. Patients were enrolled from three individual clinics within the County Emergency Hospital Cluj, and the medical team consisted of eight physicians, including an expert radiologist (R.-I.C.). The annotation process was performed using the Supervisely platform [34], where the medical team manually segmented and annotated all images using the “ADD Polygon” tool to define their contours. Annotators selected the appropriate object class, placed points along the LN boundary, and finalized the segmentation by completing the polygon. Each annotation was recorded in the Objects panel, allowing for visibility adjustments, editing, or deletion if needed. To ensure annotation accuracy and reduce subjectivity, all annotations were cross-checked and verified by the expert radiologist (R.-I.C.). For all cases included in this study, histopathology confirmation was available and approximately 85% of the US image dataset represented confirmed metastases. The most frequently scanned stations were the cervical superior (22%) and axillar lateral stations (18%), while the least represented stations were the occipital (8%) and inguinal stations (9%). Despite these variations, the dataset provided a diverse and clinically relevant sample for LN metastasis evaluation.

### 2.2. Ultrasound Examination Protocol

US examination was performed mainly using a General Electric Logiq E10s LEX101710 series Ultrasound Machine by GE Ultrasound Korea Ltd., Seongnam, South Korea. LN scans were obtained with a broadband linear transducer having the following characteristics: L2-9-D, XDclear linear probe; peripheral vascular, small parts, pediatric, abdomen, OB/GYN, general musculoskeletal, superficial musculoskeletal, neonatal, and neonatal transcranial applications; bandwidth, 2.0–10.0 MHz; field of view (max), 44 mm; physical foot print, 14 × 53 mm; penetration, up to 35 mm for the 10 MHz frequency; and acquisition mode, Thyroid1 or MSK Sup. Depth and gain were adjusted to each patient’s physical characteristics for optimized image acquisition.

Doppler-mode US was also performed (ColorFlowMetry or PowerDoppler), with the following settings: 13 color maps, including velocity variance maps; velocity scale range, 1–300 cm/s; steering angle (linear), 0–20°; auto ROI placement; and steering, linear.

Elastography-mode US was also performed for the qualitative analysis of adenopathy stiffness. The elasticity of the examined tissues could be coded in a semi-transparent color map called an elastogram. Conventionally, the scale is established as blue = hard (hard) and red = soft (soft).

In the case of patients with clinical or paraclinical/imaging suspicion of pathological adenopathy, the superficial LN stations were scanned bilaterally in the following order: 1. occipital; 2. cervical superior; 3. submandibular; 4. cervical inferior; 5. supraclavicular; 6. axillar medial; 7. axillar lateral; and 8. inguinal.

In the case of detection of pathological images of adenopathy, the previously described US equipment and settings were used for the acquisition of sets of ultrasound images: classical US, Doppler-mode US, and elastography.

### 2.3. System Architecture

**Figure 1:**
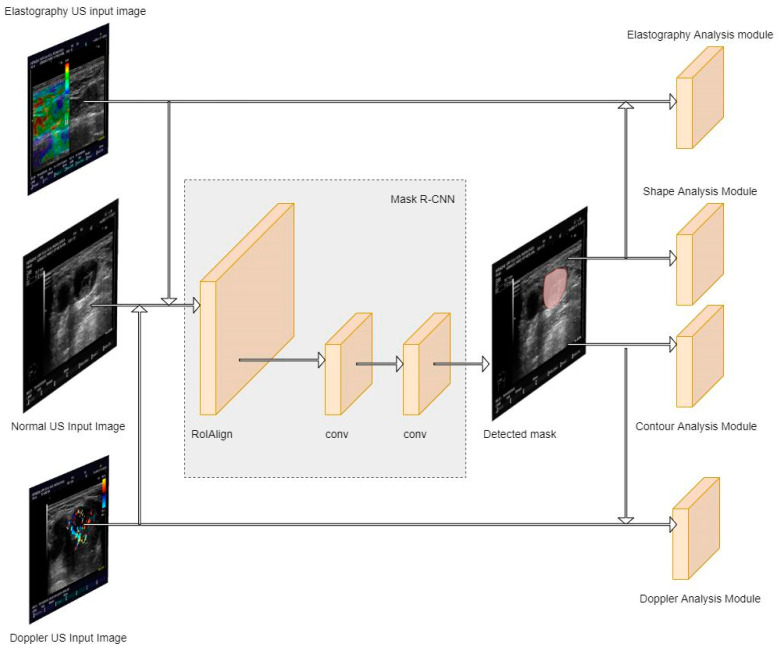
System architecture.

The proposed system of automatic detection and segmentation of multimodal data, including B-mode, Doppler, and elastography ultrasound images, for LN metastasis evaluation was based on several modules that performed analyses. The architecture of the proposed system can be observed in Figure 1. As can be observed, the system has to handle three types of input images: B-mode US, Doppler-mode US, and elastography. It contains a Mask R-CNN module responsible for LN segmentation [35] and four other analysis modules, as follows: contour analysis module and shape analysis module, responsible with determining the morphological evaluation of the analyzed LNs; Doppler analysis module, responsible for assessing the vascularity score; and elastography analysis module, responsible for assessing the tissue stiffness score.

#### 2.3.1. Detection Module

The main purpose of this module is to detect lymph nodes (LNs) in an ultrasound image (US) and find the masks of the detected LNs. The detected masks are then used by the other modules to perform further analysis for LN metastasis evaluation. The Mask R-CNN framework was chosen for LN detection and segmentation due to its ability to perform instance segmentation, which is essential for accurately delineating LN boundaries. Unlike Fast R-CNN and Faster R-CNN, which primarily generate bounding boxes, Mask R-CNN extends Faster R-CNN by incorporating a pixel-wise segmentation branch, allowing for the precise localization of LNs within ultrasound images [35]. This is particularly important in multimodal ultrasound analysis, where vascularity and stiffness evaluations depend on accurate segmentation rather than coarse bounding box approximations. Additionally, RoIAlign, an improvement over RoI pooling, enhances spatial precision by preserving finer details, which is crucial for distinguishing malignant from benign LNs [35].

In this study, two implementation versions were developed for the detection module. The first implementation of the detection module (version 1) was performed using the Detectron2 library. Detectron2 was developed by Meta AI and is built on top of PyTorch >1.8. It is the successor of Detectron and maskrcnn-benchmark. This library was chosen because it provided a fast and reliable implementation of Mask R-CNN and comparing Detectron2 with other well-known open-source Mask R-CNN implementations showed that detectron2 implementation was, in fact, faster [36,37,38,39,40].

The data used comprised 397 ultrasound (US) images of three types: B-mode US, Doppler-mode US, and elastography. The dataset was split into a training dataset (60%), a validation dataset (20%), and a test dataset (20%).

The first implementation of this module consisted of a single Mask R-CNN model for LN detection on all 3 types of US images.

Due to the limited number of US images, in order to improve the robustness of the proposed system, several data augmentation techniques (image transformations) were used on the training dataset: brightness ±25%; exposure ±10%; and blur up to 2 px. After applying augmentation, the training dataset had 1190 extra images, totaling 1429 images.

Training was performed on the Paperspace Gradient platform [37], which provides affordable cloud GPU machines which can be used to train machine learning models over a much shorter time.

For the training of the model, a custom trainer based on the DefaultTrainer provided by detectron2 [41] was used. The custom trainer was implemented based on the solution proposed in [42,43], and it added additional logic that computed the validation loss during training. The Detectron2 Model Zoo API [43] was used for trainer configuration setup, dataset usage, and evaluation frequency, which, in this case, was every ten iterations, up to the maximum number of iterations. After the training stage, the final model weights and biases were included in a file that could be exported for further use in the testing stages.

The second implementation of the detection module (version 2) was also developed, consisting of two separate models, one which was used to detect LNs in classic B-mode US and elastography, and a separate one used to detect LNs in Doppler-mode US images. This change was implemented because, for elastography images, detection is performed in the classic US part of the image. This is not possible for the Doppler-mode US images, since the vascularity information is overlaid, making it harder to detect the actual LNs. This solution was meant to improve the performance of LN detection on Doppler-mode US images. Another change considered for the second version was the use of a YOLOv8 segmentation model. While Mask R-CNN is computationally intensive, we implemented model optimization such as efficient region proposal strategies and integration with a YOLOv8 localization step to balance accuracy and processing speed, obtaining slightly better results over a shorter training time.

The first model was developed only for B-mode US and elastography image analysis. The used dataset consisted of 305 ultrasound and elastography images, split in a similar manner to the first implementation. Also, similar augmentation techniques were used, resulting in a training dataset consisting of 1098 images.

The second model was developed only for Doppler-mode US analysis. The used dataset consisted of 92 images and was split into the same categories, keeping the percentages. Finally, after the augmentation stage, the training dataset consisted of 330 images.

A comprehensive overview of the dataset’s composition, including image resolution, distribution across training, validation, and test sets, and implementation details for both versions of the detection module, is provided in Table 1, where all key information is synthesized.

#### 2.3.2. Shape Analysis Module

This module focuses on lymph node (LN) thickness, shape, and shape variability parameters for malignancy risk assessment. Essentially, it analyzes the LN contour provided by the detection module and determines the LN’s longest and shortest axes inside a minimal rectangular area surrounding the contour. The ratio of the short to long axis lengths determines the shape, while thickness is computed by multiplying the longest axis by a pixel to mm ratio, specific to every input image. The shape variability parameter is evaluated by comparing the detected LN contour to the one of a fitted ellipse. This is performed using direct ellipse fitting and shape matching with Hu moments.

While malignant or inflammatory LNs tend to be round or round–oval, a normal LN is typically oval or slightly triangular [4]. The Solbiati index (length/width ratio), with 84% accuracy, helps differentiate benign from malignant LNs: a ratio ≥ 2 suggests benignity, while a ratio ≤ 1.5 is indicative of malignancy. Additionally, capsule breaks and poorly defined contours are common in malignant LNs.

LN thickness (short diameter) is often more indicative of malignancy than length [4]. Depending on the location of the LN, the normal range of the short axis diameter vary, with a short diameter ≥10 mm being considered a potential marker for metastasis. Overall, thicker, more circular LNs and those with increased shape variability or deformation are more likely to be malignant [4,5].

#### 2.3.3. Contour Analysis Module

This module tries to quantify the contour sharpness or how well the contour of the LN is defined, i.e., how easy it would be for a human to identify the contour with an unaided eye. LNs with a poorly defined contour have a higher risk of malignancy. The proposed metric analyses the intensities of pixels, going from the inside of the contour to the outside. The description of the developed algorithm is presented in Algorithm 1. Contour sharpness evaluation.
**Algorithm 1.** Contour sharpness evaluation1:**START Contour Analysis Module** 2:       **INPUT: Mask from Mask R-CNN defining LN contour** 3:       **OUTPUT: Contour clarity metric** 4:**#** Step 1: Analyze pixels along the contour **5:       SELECT every 12th pixel along the contour for analysis** 6:# Step 2: Calculate the slope of the contour 7:       **FOR each selected pixel:** 8:    **DETERMINE slope using two neighboring pixels** 9:    # Simplify slope calculation to avoid complex computations 10:# Step 3: Draw a perpendicular line 11:       **FOR each selected pixel:** 12:    **DRAW perpendicular line to the slope through the pixel of interest** 13:    **ANALYZE a small area around the line to detect pixel intensity changes** 14:# Step 4: Measure intensity change 15:       **FOR each perpendicular line:** 16:    **CALCULATE intensity change from inside to outside the contour** 17:    **NORMALIZE intensity difference by dividing by 255** 19:# Step 5: Calculate the average intensity change 20:       **COMPUTE average intensity change across all analyzed contour pixels** 21:# Step 6: Output the metric 22:       **RETURN average intensity change as the contour sharpness metric** 23:**END Contour Analysis Module**

#### 2.3.4. Doppler Analysis Module

Global vascularity, pattern of vascular supply, and peripheral arterial resistance are important predictors of the biological nature of enlarged or otherwise suspicious LNs using Doppler-mode US images. Hence, there are different criteria that can be analyzed in order to differentiate between benign and malignant LNs [17], explained below.

1. Regarding general vascularity percentage, the vascular density is high in lymphoma and inflammatory lymph ganglia and low in physiological ones. In metastatic LNs, usually, the density of blood vessels is very low.

The vascularity percentage evaluation algorithm essentially calculates the ratio between the number of colored pixels within the detected LN contour and the total number of pixels within the mask, with a percentage below 25% indicating a possible malignant character [19]. The description of the developed algorithm is presented in Algorithm 2. Vascularity percentage evaluation.
**Algorithm 2.** Vascularity percentage evaluation1:**START Vascularity Percentage Evaluation** 2:       **INPUT: Mask from Mask R-CNN defining LN contour** 3:       **OUTPUT: Vascularity percentage** 4:# Step 1: Extract colored pixels within the mask 5:       **CONVERT the input mask to HSV color space using OpenCV** 6:# Step 2: Define HSV mask thresholds 7:       **SET lower_HSV = [0, 1, 1]** # Lower bounds: hue, saturation, value 8:       **SET upper_HSV = [180, 255, 255]** # Upper bounds: hue, saturation, value 9:# Step 3: Apply the HSV mask 10:     **COMPUTE HSV_mask by filtering pixels within the defined lower and upper bounds** 11:# Step 4: Count colored pixels 12:       **COUNT colored_pixels within the scaled mask using the HSV_mask** 13:# Step 5: Calculate vascularity percentage 14:       **COUNT total_pixels inside the scaled mask** 15:       **CALCULATE vascularity_percentage = (colored_pixels/total_pixels) × 100** 16:# Step 6: Output the result 17:       **RETURN vascularity_percentage** 18:**END Vascularity Percentage Evaluation**

2. Regarding vascular pattern/position, hilar vessels are seen in healthy LNs. Branching is not usually visible in small ones. When it comes to metastasis, the ganglia have a peripheral aberrant pattern caused by the cancerous neo-vascularization. As for inflammatory LNs, a hilar branching pattern is often found, and when it comes to lymphoma, the pattern is typically mixed and hilar, with a tree-like and extensive branching appearance.

The vascularity position evaluation algorithm tries to quantify how close the blood vessels are to the center of the LN, with a result above 0.5 indicating a possible malignant character [19], and is presented in Algorithm 3. Vascularity position evaluation.
**Algorithm 3.** Vascularity position evaluation1:**START Vascularity Position Evaluation** 2:       **INPUT: Mask defining LN contour, colored pixels identified using HSV mask** 3:       **OUTPUT: Vascularity position parameter** 4:# Step 1: Compute the centroid of the LN contour 5:       **COMPUTE centroid of LN contour** 6:# Step 2: Compute distances from colored pixels to the centroid 7:       **FOR each colored_pixel in the mask:** 8:    **CALCULATE euclidean_distance from colored_pixel to centroid** 9:    **STORE distance in colored_pixel_distances_list** 10:# Step 3: Compute the average distance for colored pixels 11:       **CALCULATE avg_colored_distance = MEAN(colored_pixel_distances_list)** 12:# Step 4: Compute distances from contour pixels to the centroid 13:       **FOR each contour_pixel in the LN contour:** 14:    **CALCULATE euclidean_distance from contour_pixel to centroid** 15:    **STORE distance in contour_pixel_distances_list** 16:# Step 5: Compute the average distance for contour pixels 17:       **CALCULATE avg_contour_distance = MEAN(contour_pixel_distances_list)** 18:# Step 6: Compute the vascularity position parameter 19:       **CALCULATE vascularity_position = avg_colored_distance/avg_contour_distance** 20:# Step 7: Output the result 21:       **RETURN vascularity_position** 22:**END Vascularity Position Evaluation**

#### 2.3.5. Elastography Analysis Module

Elasticity and stiffness of the tissue can be evaluated via elastography. A color that leans more toward the blue or red end of the spectrum, depending on the imaging technology, denotes increased stiffness and a higher chance of malignancy. Malignant lesions have higher strain ratios than benign lesions.

So, this module is focused on determining the percentage of particular color regions in elastography ultrasound images to identify tissue hardness or softness. This characteristic is important for determining lymph node (LN) malignancy, since a higher percentage of hard tissue correlates with a higher risk of malignancy. The developed algorithm essentially assesses three predefined color regions: “red”, “green”, and “blue”. This analysis is limited to the LN contour, which is why several preprocessing steps need to be performed. The elastography image usually has two parts, one with classical B-mode US and one with elastography. The LN contour is first detected on the B-mode US. Next, the detected contour is translated on the elastography part, and only then, the percentages of desired colors inside the LN contour are determined.

The red region contains two subregions defined by following HSV limits:

- Hue, 0–39; saturation, 1–255; and value, 1–255;
- Hue, 136–180; saturation, 1–255; and value, 1–255.

The green region’s HSV limits are the following: hue, 40–79; saturation, 1–255; and value, 1–255. The considered HSV limit for the blue region is the following: hue, 80–135; saturation, 1–255; and value, 1–255.

The method extracts colored pixels corresponding to each section of the translated mask and computes the percentage for each region by dividing the number of colored pixels by the total number of pixels in the mask. This approach gives quantitative data on tissue hardness, which can be used to evaluate LN malignancy. Depending on the imaging technology and heatmap settings, where blue represents hard tissue and red represents soft tissue, we established a malignancy threshold based on tissue stiffness. Specifically, if the combined percentage of blue and green pixels within the LN contour exceeds 40% in the elastography image, it is considered an indication of malignant characteristics [44,45].

Even if this translation algorithm seems simple at first glance due to the format variations in elastography images depending on the US equipment used, several iterations were needed before a robust solution could be achieved.

The first version assumed that the B-mode US part was always on the right side of the image and the elastography part was on the left. This assumption proved to be erroneous when the input images came from multiple and different models of US equipment. The final version of the translation algorithm was more complex and included two sub-algorithms, one for the detection of the B-mode US region and one for detection of the colored elastography region. All algorithms are detailed in Algorithm 4. Translation algorithm.
**Algorithm 4.** Translation algorithm1:**START Translate Contour** 2:       **INPUT: Image, Contour** 3:       **OUTPUT: Translated Contour** 4:# Step 1: Detect and remove the color scale 5:       **CALL DetectAndRemoveColorScale(image)** 6:# Step 2: Detect the region containing the actual ultrasound (US) image 7:       **ultrasound_region = DetectUltrasoundRegion(image)** 8:# Step 3: Detect the colored region to determine translation direction 9:       **colored_region_side = DetectColoredRegion(image)** 10:# Step 4: Translate the contour 11:       **IF colored_region_side == “left”:** 12:    **FOR each pixel in contour:** 13:           **TRANSLATE pixel_x = pixel_x + (ultrasound_region.width/2)** 14:       **ELSE:** 15:    **FOR each pixel in contour:** 16:           **TRANSLATE pixel_x = pixel_x − (ultrasound_region.width/2)** 17:       **END IF** 18:       **RETURN Translated Contour** 19:**END TranslateContour**20:# Detect B-mode US Region Algorithm 21:# Identifies the region containing the US image, excluding margins. 22:**START Detect Ultrasound Region** 23:       **INPUT: Image** 24:       **OUTPUT: Ultrasound Region** 25:# Step 1: Preprocessing 26:       **APPLY bilinear_filter to image to reduce noise** 27:       **APPLY morphological_opening to remove noise outside main object** 28:       **BINARIZE image using a threshold** 29:# Step 2: Find connected components 30:       **COMPONENTS = connectedComponentsWithStats(binarized_image)** 31:# Step 3: Identify the largest connected component 32:       **ultrasound_region = FIND largest connected component in COMPONENTS** 33:       **RETURN ultrasound_region** 34:**END Detect Ultrasound Region**35:# Detect Colored Elastography Region Algorithm 36:# Determines whether the elastography region is on the left or right side of the image. 37:**START Detect Colored Region** 38:       **INPUT: Image** 39:       **OUTPUT: Side of the image with more colored pixels (“left” or “right”)** 40:# Step 1: Convert image to HSV color space 41:       **hsv_image = CONVERT image to HSV** 42:# Step 2: Extract colored pixels in the specified range 43:       **colored_pixels = EXTRACT pixels in range (0, 10, 10) to (180, 255, 255)** 44:# Step 3: Count colored pixels in left and right halves of the image 45:       **left_count = COUNT colored_pixels in left half of hsv_image** 46:       **right_count = COUNT colored_pixels in right half of hsv_image** 47:# Step 4: Determine the side with more colored pixels 48:       **IF left_count > right_count:** 49:    **RETURN “left”** 50:       **ELSE:** 51:    **RETURN “right”** 52:       **END IF** 53:**END Detect Colored Region**

### 2.4. Detection Module Evaluation

This section focuses on the testing methods of the detection model and each individual image processing module and presents the evaluation procedure and results.

The various implementations of the detection model were carefully evaluated both during and after training in order to ensure a robust performance. This is why the available dataset was always split into a training dataset (60%), a validation dataset (20%), and a test dataset (20%). During the evaluation period, several key metrics were considered; these are explained below.

#### 2.4.1. Accuracy Metrics on the Training Dataset

The model’s accuracy on the training dataset was assessed using a conventional classification algorithm based on false positives (*FP*s), false negatives (*FN*s), true positives (*TP*s), and true negatives (*TN*s). The formula for accuracy is as follows:

(1) $$Accuracy=\frac{TN+TP}{TN+TP+FN+FP}$$

#### 2.4.2. Total Loss on the Training Dataset

The total_loss is a weighted sum of individual losses computed during the iteration. By default, the weights are one. The individual losses are as follows:

○ loss_cls: Classification loss in the ROI head [35], determines how effectively the model labels a predicted box with the appropriate class;
○ loss_box_reg: Localization loss in the ROI head [35], calculates the box localization loss (predicted location vs. true location);
○ loss_rpn_cls: Classification loss in the region proposal network (RPN) [35], evaluates how effectively the RPN labels the anchor boxes as foreground or background.
○ loss_rpn_log: Localization loss in the RPN [35], calculates the RPN’s localization loss for the predicted regions;
○ loss_mask: Mask loss in the Mask head [35], identifies the correctness of the predicted binary masks.

#### 2.4.3. AP Metrics for Segmentation Tasks on Validation and Test Datasets

The AP metrics were computed using the COCO evaluator [46] provided by detectron2. AP (average precision) is a popular metric in measuring the accuracy of object detectors. To compute the AP, first, the precision and recall are computed using the following formulas:

(2) $$Precision=\frac{TP}{TP+FP}$$

(3) $$Recall=\frac{TP}{TP+FN}$$

To classify whether a prediction is *TP* or *FP*, the intersection over union (IoU) is computed (also known as the Jaccard index). Next, the precision against the recall plot is computed for a given threshold for IoU. The general definition for the AP is finding the area under the precision–recall curve [33]. For the COCO implementation, the AP is the average over multiple IoUs, specifically for IoUs from 0.5 to 0.95 with a step size of 0.05. AP50 is for an IoU of 0.5, and AP75 is for an IoU of 0.75. Moreover, APs, APm, and APl are for small, medium and large objects, respectively [47].

#### 2.4.4. Loss on Validation Dataset

The validation_loss was computed so that training could be stopped before the model started overfitting to the training data. The validation_loss was computed in the same way as the total_loss but on the validation dataset.

## 3. Results

This section focuses on the testing methods of the detection model and each individual image processing module and presents the evaluation procedure and results.

### 3.1. Detection Module Performance Evaluation

As presented in the previous section, this module presented two implementation versions, developed to achieve improved performance. Both implementations were evaluated using the same procedure.

**Figure 2:**
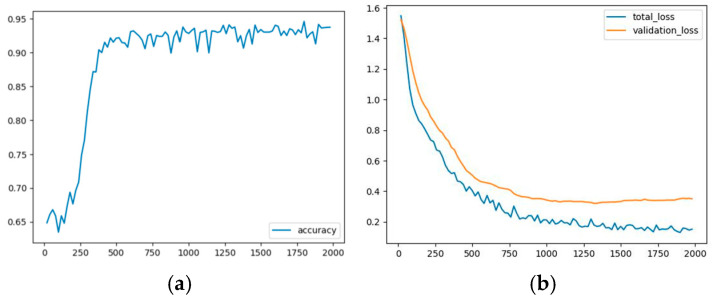
(**a**) Accuracy evolution during training. The *x*-axis represents the number of iterations, and the *y*-axis represents the accuracy. (**b**) Loss measures’ evolution during training. The *x*-axis represents the number of iterations, and the *y*-axis represents the loss.

Table 2 presents all the metrics registered during the last iteration on the train dataset for the first implementation. The observed accuracy was 92.56%, and the total_loss was 0.1453. Moreover, Figure 2a presents the evolution of the accuracy during the training process, while Figure 2b presents the evolution of both the validation and the total losses during the 2000 iterations for which the model was run. The model started overfitting somewhere around iteration 1200.

The AP metrics on the validation dataset computed during the last iteration of the training process and for the test dataset computed with the trained model are presented in Table 3.

The second version of the detection module consisted of two separate models, one to detect LNs on B-mode US and elastography—the classic US model—and one to detect LNs on Doppler-mode US, the Doppler US model.

**Figure 3:**
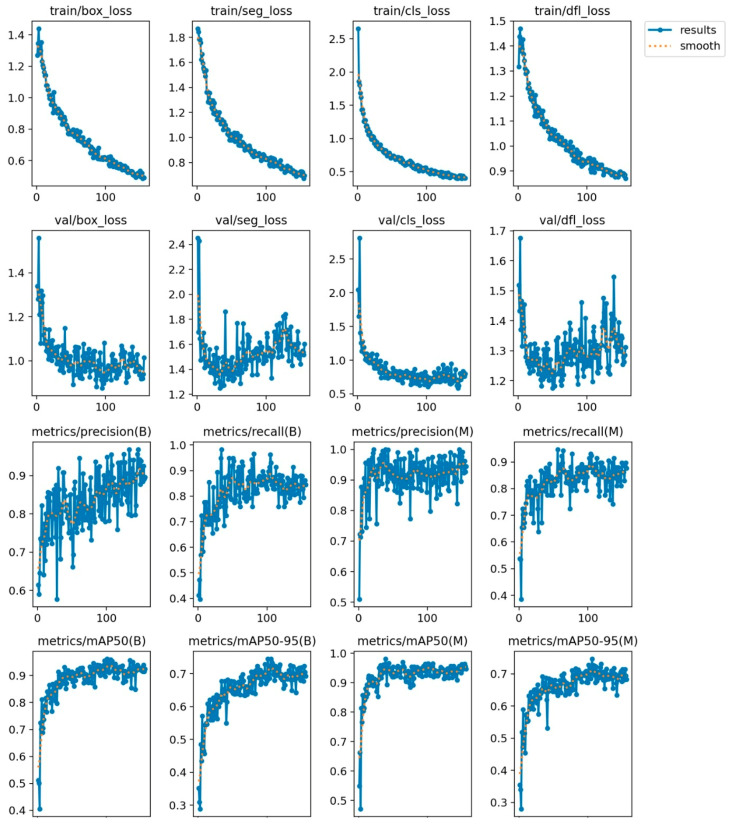
Accuracy metrics during training for the classic US model.

The training of the classic US model was scheduled for 300 epochs, but it stopped early (after 156 epochs) as no improvement was observed for 50 epochs. The best results were observed at epoch 106, and that is the model that was used for inference. The metrics registered during training can be seen in Figure 3 below.

The AP metrics on the validation dataset and for the test dataset computed with the trained model are presented in Table 4.

**Figure 4:**
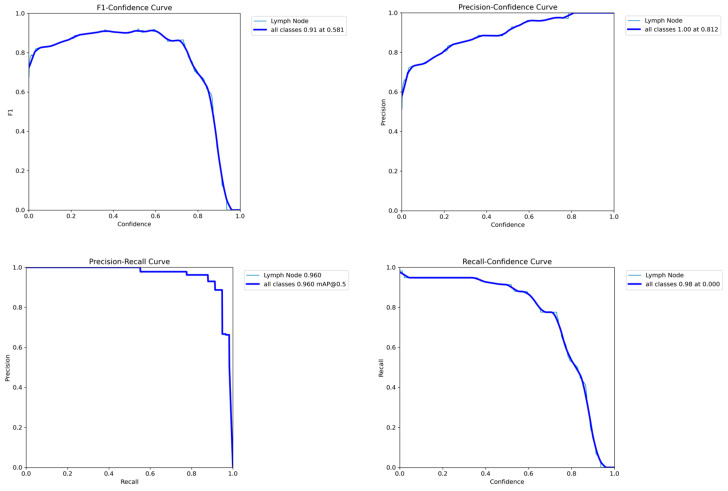
Performance evaluation of the classic US model on the validation dataset.

Figure 4 presents performance metrics like the F1–confidence curve, the precision–confidence curve, the precision–recall curve, and the recall–confidence curve for the classic US model on the validation dataset.

**Figure 5:**
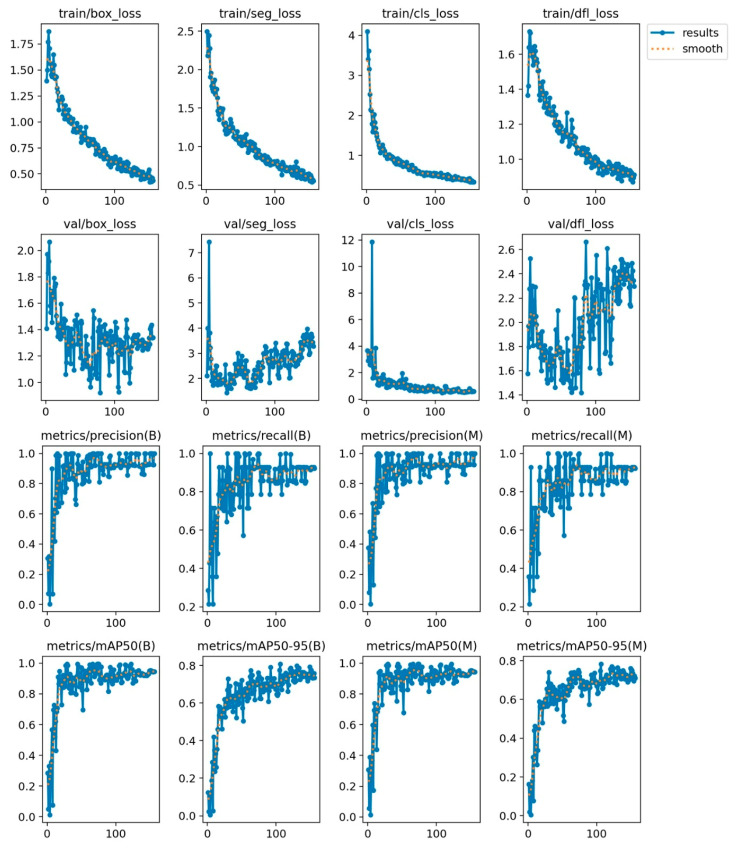
Accuracy metrics during training for the Doppler US model.

The training of the Doppler US model was scheduled for 200 epochs, but it stopped early (after 156 epochs) as no improvement was observed for 50 epochs. The best results were observed at epoch 106, and that is the model that was used for inference. The metrics registered during training can be seen in Figure 5 below.

The AP metrics on the validation dataset were also evaluated and are presented in Table 5, together with the AP metrics for the test dataset computed with the trained model.

**Figure 6:**
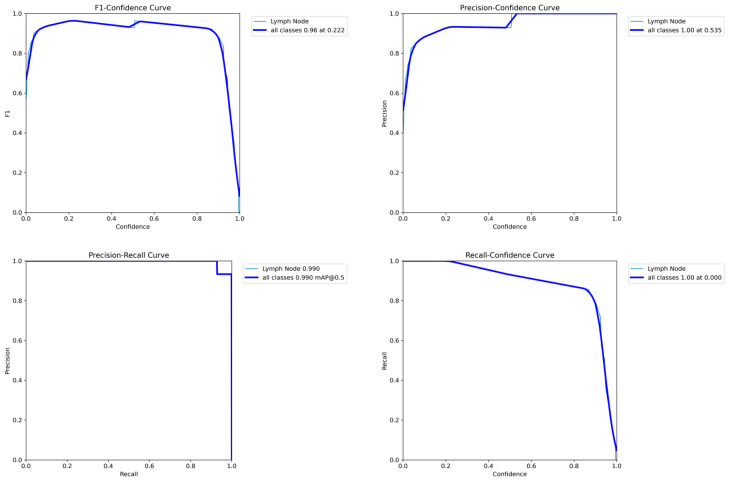
Performance evaluation of the Doppler US model on the validation dataset.

Figure 6 presents the performance metrics for the Doppler US model. Similar performances, F1 score, high precision, and recall were achieved.

### 3.2. Shape Analysis Module and Contour Analysis Module on B-Mode US Performance Evaluation

**Figure 7:**
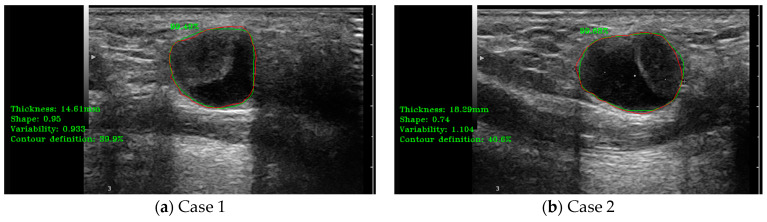

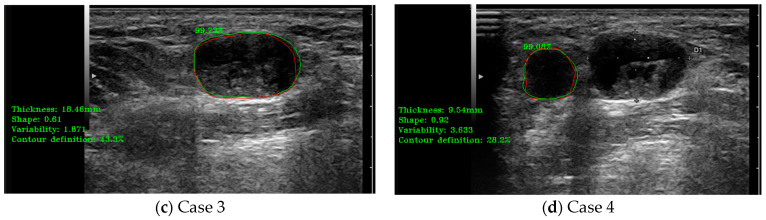
(**a**–**d**) illustrate cases of lymph node (LN) automatic detection using B-mode ultrasound (US) and shape parameter evaluation. The red contour represents the manually delineated region by the doctor, while the green contour corresponds to the system’s automatically detected LN boundary. The percentage value indicates the detection accuracy.

The detection results obtained using the final version of the detection module and the main shape parameters results, including thickness, length, and area, shape form (elliptical vs. circular), shape variability, and the main contour analysis parameter (contour sharpness/definition), are shown in Figure 7.

### 3.3. Doppler Analysis Module Performance Evaluation

**Figure 8:**
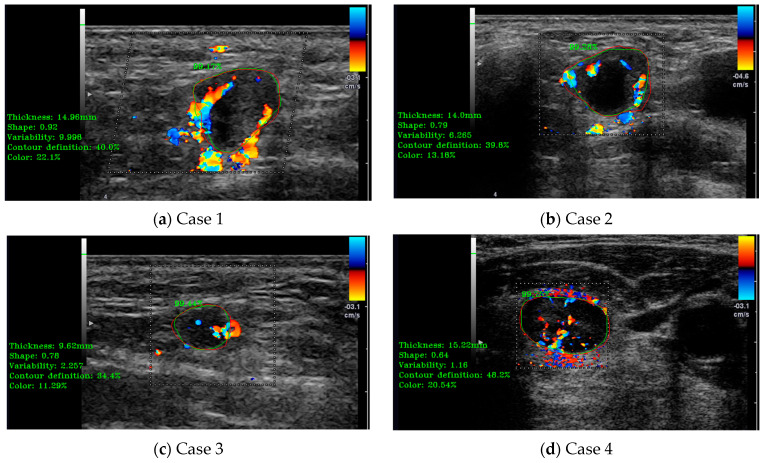
(**a**–**d**) illustrate cases of lymph node (LN) automatic detection using Doppler-mode ultrasound (US), incorporating shape parameter evaluation and Doppler US indicator assessment for enhanced accuracy. The red contour represents the manually delineated region by the doctor, while the green contour corresponds to the system’s automatically detected boundary. The percentage value indicates the detection accuracy.

In this case, the medical indicators for B-mode US evaluation that were also valid for Doppler-mode US were kept. Besides those, some specific indicators and algorithms were developed. The specific Doppler ultrasound indicators evaluated some extra parameters, such as the vascularity ratio and the vascularity position presented in Figure 8. On the left side of the images are printed, in a green color, the numerical results of several indicators, including the vascularity ratio, in the percentages named “Color”. As can be seen for all the cases presented in Figure 8, the percentages are below the defined cut-off threshold for metastasis indication.

### 3.4. Elastography Analysis Module Performance Evaluation

**Figure 9:**
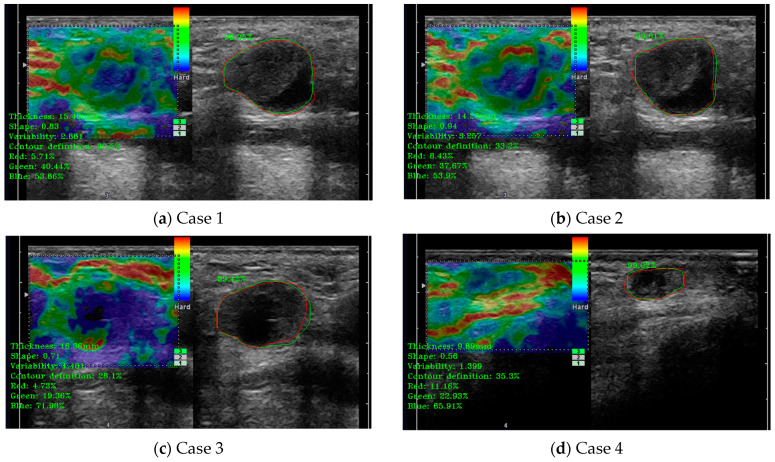
(**a**–**d**) illustrate cases of lymph node (LN) automatic detection using elastography, integrating shape parameter evaluation and elastography color indicator assessment for improved diagnostic precision. The red contour represents the manually delineated region by the doctor, while the green contour corresponds to the system’s automatically detected boundary. The percentage value indicates the detection accuracy.

In this case, the medical indicators for B-mode US evaluation that were also valid for elastography were kept. Besides those, some specific indicators and algorithms were developed. The specific elastography indicators evaluated some extra parameters like tissue rigidity. The two sub-algorithms for the detection of the B-mode US region and the colored elastography region were also evaluated; the results are presented in Figure 9. In a similar manner to the Doppler analysis module, on the left side of the images are printed, in a green color, the numerical results of several indicators, including the percentage of the analyzed color classes “Red”, “Green”, and “Blue”. For all the cases presented in Figure 9, the combined percentage of “green” and “blue” was above the defined cut-off threshold for metastasis.

## 4. Discussion

This work presents a complex DL-based automated system for LN detection and segmentation using multimodal US imaging, including a B-mode, a Doppler mode, and elastography. The developed system was evaluated using two implementations of the detection module to enhance performance across different ultrasound imaging modalities. The Mask R-CNN approach presented good segmentation capabilities, with COCO AP scores of 64 on the validation dataset and 60.7 on the test dataset, with an accuracy of 92.56% on the training dataset. However, to obtain better segmentation results on the Doppler-mode images, a two-model approach was used, resulting in improved accuracy. By allowing a thorough study of vascularity and stiffness parameters, the integration of Doppler and elastography data improved the diagnostic capacities of the system even further. These modifications highlighted LN shape, vascular patterns, and tissue hardness, therefore addressing important gaps in present diagnostic techniques.

A key strength of this study is that all included LN cases had histopathological confirmation, ensuring reliable ground truth labels for both benign and metastatic cases. While the dataset was not perfectly balanced across different cancer types or LN stations, the distribution remained reasonably proportional and did not affect the primary study objective, which was to detect LN modifications indicative of metastasis, rather than classifying specific cancer types.

Data augmentation techniques were applied to improve model robustness and reduce overfitting. Additionally, future work will expand the dataset with multi-center data to improve generalizability across diverse populations and imaging conditions. The variability in ultrasound equipment already posed a challenge at this stage, requiring robust preprocessing techniques to handle image format differences.

The Supervisely platform was used for manual LN segmentation and annotation, with all annotations cross-checked and verified by an expert radiologist (R.I.C.). While this step minimized subjectivity, multi-expert validation could further improve reproducibility and reliability. Since vascularity and elastography analyses depend on precise contour delineation, segmentation errors could impact downstream results. To address this, future work will explore automated annotation refinement, including autoencoders, uncertainty-based correction, and unsupervised learning methods, to enhance segmentation consistency and dataset quality.

Elastography imaging provides valuable information on tissue stiffness, a key malignancy indicator. Besides describing the color hue scale, a specific cut-off threshold was also defined in our study for distinguishing benign and malignant LNs. Clinical studies indicate that a higher stiffness (blue–green-dominant regions) suggests malignancy, while softer tissue (red-dominant) indicates benign conditions. In this study, a 40% blue–green ratio within the LN contour was used, aligning with histopathological findings. Future research will explore machine learning-based threshold optimization to further refine these criteria.

While this study demonstrates the feasibility and accuracy of an automated DL-based system for LN metastasis evaluation, it also lays the foundation for future clinical integration and validation. To this end, several additional algorithms were developed to manage image format variation in terms of resolution but also due to different types of US equipment used, which will be the focus of a different study. The experimental results show that the system is capable of accurately detecting and segmenting LN from multimodal ultrasound images, providing valuable insights into LN morphology, vascularity, and stiffness. These results suggest that the system could be used as a decision-support tool for clinicians, reducing operator dependency and standardizing LN assessments. In clinical practice, this could enhance early detection, improve treatment planning, and enable more precise patient stratification. It could be integrated into ultrasound (US) equipment software, providing real-time assistance to clinicians during patient evaluations. Alternatively, the system could function as an independent tool, either locally or cloud-based, enabling physicians to analyze and track LN changes over time using stored ultrasound images from previous evaluations. This flexibility allows for seamless integration into existing workflows, enhancing diagnostic consistency and longitudinal monitoring.

Furthermore, this DL-driven approach has potential applications in robotic-assisted oncologic surgery. As demonstrated by Rus et al., real-time, high-precision diagnostic insights could reduce intraoperative risks and improve surgical decision making [48]. Additionally, Tucan et al. explored its use in brachytherapy for non-resectable liver tumors, where non-invasive preoperative assessments could improve treatment planning and patient outcomes [49].

Although the system’s performance is promising, there are still areas for improvement, such as the expansion of the dataset by including multiple centers, the refinement of the annotation techniques, and real-time optimization strategies such as pruning and quantization to bridge the gap between experimental findings and practical implementation. Future studies will involve hybrid imaging modalities such as PET-CT to augment ultrasound-based analysis and clinical validation trials to assess real-world applicability.

## 5. Conclusions

An important development in automated medical diagnostics is provided by the suggested approach for evaluating LN metastases utilizing DL and multimodal ultrasound imaging. The second implementation’s addition of two specialized detection models is a significant advancement that tackles the difficulties of Doppler image analysis and improves segmentation accuracy in all modalities.

The developed system proved to be highly accurate, efficient, and capable of providing real-time, non-invasive LN analysis. The method offers a thorough evaluation of LN characteristics by integrating sophisticated elements like vascularity and stiffness evaluation, which lowers diagnostic variability and facilitates individualized treatment planning. The flexibility and potential of this system in various clinical contexts are further demonstrated by the second implementation, which takes a customized approach for various imaging modalities. 

## Figures and Tables

**Table 1:** Detection module implementation details and dataset information.

	Detection Module—Version 1	Detection Module—Version 2	
Model No.	Unique Model	US Model	Doppler US Model
Implementation	Detecton2 implementation of Mask R-CNN	YOLOv8 implementation of Mask R-CNN	YOLOv8 implementation of Mask R-CNN
Image type	B-US, D-US, E-US *	B-US, E-US *	D-US *
Dataset	397 images	305 images	92 images
Augmented dataset	2382 images	1830 images	552 images
Image resolution	760 × 574 1280 × 876 1442 × 802 1552 × 873	760 × 574 1280 × 876 1442 × 802 1552 × 873	760 × 574 1280 × 876 1442 × 802 1552 × 873
Training dataset (60%)	1428 images	1098 images	330 images
Validation dataset (20%)	477 images	366 images	111 images
Test dataset (20%)	477 images	366 images	111 images

* Abbreviations: B-US = B-mode ultrasound; D-US = Doppler-mode ultrasound; and E-US = elastography.

**Table 2:** Evaluation metrics during training for the first version of the detection module.

Metrics	Value
fast_rcnn/cls_accuracy	0.98828125
fast_rcnn/false_negative	0.1788537549
fast_rcnn/fg_cls_accuracy	0.8211462451
loss_box_reg	0.02857693098
loss_cls	0.01522202883
loss_mask	0.09584703296
loss_rpn_cls	0.00001277672754
loss_rpn_loc	0.001906370104
mask_rcnn/accuracy	0.9256435529
mask_rcnn/false_negative	0.06005441454
mask_rcnn/false_positive	0.05255943234
roi_head/num_bg_samples	494
roi_head/num_fg_samples	18
rpn/num_neg_anchors	252
rpn/num_pos_anchors	4
total_loss	0.1452940319

**Table 3:** Segmentation performance for the first version of the detection module.

Metrics	Test Dataset	Validation Dataset
AP	60.6610	64.0297
AP50	88.2196	89.9009
AP75	63.5684	77.2141

**Table 4:** Segmentation performance for the second version of the detection module (classical US model).

Metrics	Test Dataset	Validation Dataset
AP	75.0419	74.8861
AP50	92.5031	95.8426
AP75	86.8613	87.2800

**Table 5:** Segmentation performance for the second version of the detection module (Doppler US model).

Metrics	Test Dataset	Validation Dataset
AP	82.8358	82.8358
AP50	99.0333	99.0333
AP75	98.6250	98.6250

## Data Availability

The original contributions presented in this study are included in the article. Further inquiries can be directed to the corresponding author(s).
